# Supplementary material for: Global, regional, and national burdens of Clostridioides difficile infection over recent decades: a trend analysis informed by the Global Burden of Disease Study
Source: Microbiol Spectr. 2025 Apr 24;13(6):e01290-24. doi: 10.1128/spectrum.01290-24 (PMC12131777; doi:10.1128/spectrum.01290-24)

Supplementary Materials

Supplementary Table1. AAPC of ASDR and ASMR in different age groups across 5 SDI regions from 1990 to 2019

Age (years)	Global		High SDI		High-middle SDI		Middle-SDI		Low-middle SDI		Low SDI	
	ASDR, ratio (95% CI)	ASMR, ratio (95% CI)	ASDR, ratio (95% CI)	ASMR, ratio (95% CI)	ASDR, ratio (95% CI)	ASMR, ratio (95% CI)	ASDR, ratio (95% CI)	ASMR, ratio (95% CI)	ASDR, ratio (95% CI)	ASMR, ratio (95% CI)	ASDR, ratio (95% CI)	ASMR, ratio (95% CI)
**0 - 14**	0.34 (-0.02 - 0.70)	0.31 (-0.04 - 0.66)	-0.51 (-0.80 - -0.22)	-0.51 (-0.80 - -0.22)	-0.03 (-0.54 - 0.48)	-0.08 (-0.58 - 0.42)	1.47 (1.20 - 1.74)	1.42 (1.16 - 1.69)	0.70 (0.19 - 1.22)	0.68 (0.17 - 1.19)	-1.71 (-2.04 - -1.37)	-1.70 (-2.03 - -1.36)
**15 - 44**	0.38 (-0.01 - 0.77)	0.45 (0.06 - 0.85)	1.58 (1.37 - 1.79)	1.71 (1.49 - 1.94)	-0.02 (-0.23 - 0.26)	0.10 (-0.16 - 0.36)	0.91 (0.52 - 1.30)	0.10 (-0.16 - 0.36)	0.54 (0.32 - 0.77)	0.60 (0.39 - 0.81)	-1.49 (-1.66 - -1.33)	-1.46 (-1.62 - -1.30)
**45 - 69**	2.86 (2.71 - 3.00)	3.03 (2.88 - 3.19)	4.31 (4.01 - 4.62)	4.42 (4.09 - 4.74)	2.35 (1.95 - 2.74)	2.54 (2.22 - 2.85)	1.84 (1.45 - 2.23)	1.99 (1.64 - 2.34)	1.22 (0.95 - 1.50)	1.29 (1.01 - 1.58)	-1.11 (-1.36 - -0.86)	-1.04 (-1.29 - -0.78)
**70 - 95**	4.23 (3.92 - 4.54)	4.18 (3.98 - 4.38)	4.98 (4.65 - 5.31)	4.89 (4.55 - 5.23)	4.30 (3.78 - 4.81)	4.34 (3.82 - 4.86)	3.78 (3.52 - 4.03)	3.86 (3.56 - 4.17)	2.49 (2.17 - 2.82)	2.65 (2.32 - 2.99)	0.12 (-0.34 - 0.58)	0.27 (-0.24 - 0.78)
**＜5**	0.51 (0.09 - 0.93)	0.49 (0.07 - 0.90)	-0.55 (-0.85 - -0.25)	-0.56 (-0.86 - -0.26)	0.20 (-0.22 - 0.62)	0.16 (-0.26 - 0.58)	1.73 (1.36 - 2.09)	1.71 (1.39 - 2.03)	0.85 (0.68 - 1.02)	0.84 (0.67 - 1.00)	-1.73 (-2.16 - -1.29)	-1.72 (-2.15 - -1.28)

Note: AAPC: average annual percentage changes; SDI: socio-demographic index; ASDR: age-standardized disability-adjusted life year; ASMR: age-standardized mortality rate.

Supplementary Table2. The join-points of ASMR and ASDR in different SDI regions from 1990 to 2019.

Age	Measure	Location	Years Join-point 1<br>APC (95%CI)	Years Join-point 2<br>APC (95%CI)	Years Join-point 3<br>APC (95%CI)	Years Join-point 4<br>APC (95%CI)	Years Join-point 5<br>APC (95%CI)
ALL ages	ASMR	Global	1990-1997***<br>2.78 (2.47 to 3.09)	1997-2003***<br>8.43 (7.98 to 8.88)	2003-2008***<br>5.06 (4.49 to 5.64)	2008-2016<br>-0.21 (-0.44 to 0.02)	2016-2019***<br>-3.39 (-4.23 to -2.55)
		High SDI	1990-1997***<br>4.18 (3.92 to 4.43)	1997-2003***<br>12.42 (11.97 to 12.87)	2003-2007***<br>8.12 (7.09 to 9.15)	2007-2016*<br>0.26 (0.05 to 0.47)	2016-2019***<br>-3.75 (-4.65 to -2.83)
		High-middle SDI	1990-1996***<br>1.74 (1.12 to 2.35)	1996-2012***<br>2.86 (2.74 to 2.98)	2012-2016<br>0.66 (-0.55 to 1.87)	2016-2019*<br>-1.37 (-2.53 to -0.2)	-
		Middle SDI	1990-2012***<br>1.83 (1.74 to 1.92)	2012-2019<br>0.26 (-0.17 to 0.69)	-	-	-
		Low-middle SDI	1990-1992<br>1.92 (-0.62 to 4.51)	1992-1995<br>-2.12 (-4.6 to 0.43)	1995-2005<br>-0.14 (-0.36 to 0.09)	2005-2013***<br>2.38 (2.05 to 2.71)	2013-2019***<br>1.79 (1.4 to 2.18)
		Low SDI	1990-1993<br>-0.69 (-2.33 to 0.97)	1993-2005***<br>-2.85 (-3.09 to -2.62)	2005-2015***<br>-0.75 (-1.09 to -0.4)	2015-2019<br>1.02 (-0.19 to 2.24)	-
	ASDR	Global	1990-1997***<br>1.25 (0.86 to 1.65)	1997-2003***<br>3.45 (2.88 to 4.03)	2003-2008***<br>2.70 (2.05 to 3.36)	2008-2013<br>0.25 (-0.35 to 0.86)	2013-2019***<br>-0.93 (-1.25 to -0.61)
		High SDI	1990-1997***<br>1.7 (1.39 to 2.01)	1997-2005***<br>8.48 (8.23 to 8.74)	2005-2008***<br>4.58 (3 to 6.19)	2013-2016<br>-0.07 (-0.26 to 0.13)	2016-2019***<br>-2.85 (-3.59 to -2.11)
		High-middle SDI	1990-1999***<br>1.77 (1.38 to 2.16)	1999-2012***<br>0.41 (0.2 to 0.62)	2012-2019**<br>-0.63 (-1.03 to -0.22)	-	-
		Middle SDI	1990-1992**<br>3.95 (1.03 to 6.95)	1992-2012***<br>1.6 (1.52 to 1.69)	2012-2017<br>-0.57 (-1.33 to 0.19)	2017-2019<br>1.93 (-0.35 to 4.26)	-
		Low-middle SDI	1990-1992<br>1.75 (-1.44 to 5.05)	1992-1995<br>-2.4 (-5.61 to 0.92)	1995-2005*<br>-0.31(-0.61 to -0.01)	2005-2019***<br>2.05 (1.91 to 2.2)	-
		Low SDI	1990-1993<br>-0.77 (-2.51 to 1.01)	1993-2005***<br>-3.07 (-3.32 to -2.82)	2005-2015***<br>-1.21 (-1.58 to -0.83)	2015-2019<br>1.31 (-0.02 to 2.67)	-
<5 years	ASMR	Global	1990-1992<br>2.97 (-0.55 to 6.62)	1995-2005<br>0.13 (-0.07 to 0.33)	2005-2013**<br>0.66 (0.24 to 1.08)	2013-2016<br>-1.26 (-4.2 to 1.78)	2016-2019*<br>1.69 (0.22 to 3.18)
		High SDI	1990-2000***<br>-1.01 (-1.31 to -0.71)	2000-2007***<br>1.67 (1.12 to 2.23)	2007-2011**<br>-2.47 (-3.82 to -1.1)	2011-2016<br>-0.37 (-1.19 to 0.45)	2016-2019**<br>-2.51 (-3.81 to -1.19)
		High-middle SDI	1990-1999***<br>2 (1.41 to 2.6)	1999-2009<br>-0.12 (-0.61 to 0.37)	2009-2017***<br>-1.9 (-2.54 to -1.25)	2017-2019<br>1.65 (-3.1 to 6.63)	-
		Middle SDI	1990-1992**<br>4.94 (1.45 to 8.55)	1992-2012***<br>1.85 (1.76 to 1.95)	2012-2017<br>-0.57 (-1.43 to 0.29)	2017-2019*<br>2.9 (0.32 to 5.54)	-
		Low-middle SDI	1990-2005***<br>-0.72 (-0.96 to -0.48)	2005-2019***<br>2.53 (2.27 to 2.79)	-	-	-
		Low SDI	1990-1992<br>-0.04 (-4.31 to 4.43)	1992-2006***<br>-3.17 (-3.42 to -2.91)	2006-2016***<br>-1.41 (-1.91 to -0.9)	2016-2019*<br>3.04 (0.2 to 5.97)	-
	ASDR	Global	1990-1992<br>2.96 (-0.59 to 6.64)	1995-2005<br>0.15 (-0.05 to 0.36)	2005-2013**<br>0.68 (0.26 to 1.11)	2013-2016<br>-1.25 (-4.22 to 1.81)	2016-2019*<br>1.73 (0.25 to 3.24)
		High SDI	1990-2000***<br>-1.01 (-1.31 to -0.7)	2000-2007***<br>1.66 (1.1 to 2.22)	2007-2011**<br>-2.49 (-3.85 to -1.1)	2011-2016<br>-0.35 (-1.17 to 0.48)	2016-2019**<br>-2.49 (-3.8 to -1.16)
		High-middle SDI	1990-1999***<br>2.05 (1.46 to 2.64)	1999-2009<br>-0.08 (-0.57 to 0.41)	2009-2017***<br>-1.9 (-2.53 to -1.25)	2017-2019<br>1.76 (-2.97 to 6.71)	-
		Middle SDI	1990-1992**<br>5.08 (1.68 to 8.6)	1992-2013***<br>1.85 (1.76 to 1.93)	2013-2016<br>-1.73 (-4.34 to 0.96)	2016-2019**<br>2.2 (0.93 to 3.48)	
		Low-middle SDI	1990-2005***<br>-0.71 (-0.95 to -0.47)	2005-2019***<br>2.55 (2.29 to 2.81)	-	-	-
		Low SDI	1990-1992<br>-0.04 (-4.33 to 4.44)	1992-2006***<br>-3.18 (-3.43 to -2.92)"	2006-2016***<br>-1.42 (-1.92 to -0.91)"	2016-2019*<br>3.05 (0.2 to 5.98)	-
>70 years	ASMR	Global	1990-1997***<br>4.77 (4.46 to 5.09)	1997-2002***<br>13.45 (12.71 to 14.21)	2002-2007***<br>8.04 (7.32 to 8.76)	2007-2016<br>-0.02 (-0.26 to 0.22)	2016-2019***<br>-4.68 (-5.73 to -3.61)
		High SDI	1990-1997***<br>5.58 (5.25 to 5.91)	1997-2002***<br>14.93 (14.17 to 15.71)	2002-2007***<br>9 (8.26 to 9.73)	2007-2016<br>0.09 (-0.15 to 0.32)	2016-2019***<br>-3.91 (-4.97 to -2.85)
		High-middle SDI	1990-1997**<br>1.15 (0.58 to 1.73)	1997-2004***<br>9.23 (8.44 to 10.02)	2004-2011***<br>6.79 (6 to 7.58)	2011-2016***<br>3.09 (1.61 to 4.6)	2016-2019*<br>-2.73 (-4.96 to -0.44)
		Middle SDI	1990-1996***<br>1.35 (0.9 to 1.79)	1996-2005***<br>3.32 (3.03 to 3.62)	2005-2009***<br>8.3 (6.91 to 9.72)	2009-2014***<br>4.74 (3.89 to 5.6)	2014-2019***<br>3.49 (2.9 to 4.09)
		Low-middle SDI	1990-1999***<br>1.47 (1.08 to 1.86)	1999-2011***<br>3.51 (3.25 to 3.77)	2011-2017***<br>4.3 (3.44 to 5.17)	2017-2019<br>-1.96 (-5.59 to 1.81)	-
		Low SDI	1990-2006***<br>-0.55 (-0.78 to -0.33)	2006-2012***<br>2.97 (1.72 to 4.24)	2012-2017<br>1.13 (-0.5 to 2.79)	2017-2019<br>-3.24 (-8.34 to 2.14)	-
	ASDR	Global	1990-1997***<br>4.49 (4.2 to 4.77)	1997-2002***<br>13.43 (12.77 to 14.09)	2002-2007***<br>8.17 (7.56 to 8.79)	2007-2016<br>-0.12 (-0.32 to 0.08)	2016-2019***<br>-3.89 (-4.79 to -2.97)
		High SDI	1990-1997***	1997-2002***	2002-2007***	2007-2015	2015-2019***

	5.42 (5.09 to 5.75)	15.15 (14.41 to 15.91)	9.19 (8.51 to 9.89)	0.08 (-0.19 to 0.34)	-2.61 (-3.24 to -1.98)
High-middle	1990-1997**	1997-2004***	2004-2012***	2012-2016*	2016-2019*
SDI	1.06 (0.5 to 1.62)	8.79 (8.03 to 9.56)	6.57 (5.98 to 7.17)	2.92 (0.75 to 5.14)	-2.39 (-4.47 to -0.27)
Middle SDI	1990-1996***	1996-2005***	2005-2009***	2009-2014***	2014-2019***
	1.41 (0.99 to 1.84)	3.25 (2.97 to 3.53)	8.2 (6.87 to 9.54)	4.51 (3.7 to 5.33)	3.4 (2.84 to 3.97)
Low-middle SDI	1990-2000***	2000-2011***	2011-2017***	2017-2019	-
	1.18 (0.86 to 1.5)	3.28 (2.98 to 3.57)	4.62 (3.78 to 5.47)	-1.44 (-5.01 to 2.26)	
Low SDI	1990-2006***	2006-2022***	2022-2027*	2017-2019	-
	-1.08 (-1.27 to -0.88)	3.16 (2.03 to 4.29)	1.46 (0.01 to 2.94)	-2.54 (-7.12 to 2.27)	

Note: SDI: socio-demographic index; ASMR: age-standardized mortality rate; ASDR: age-standardized disability-adjusted life rate; APC: Annual percentage change; * $P < 0.05$, ** $P < 0.01$, *** $P < 0.001$.

Supplement Figure Legends

Supplementary Figure S1. The correlation between SDI and ASR in 21 regions and 204 countries. The correlation between SDI and ASDR in 21 regions (A) and 204 countries (C). The correlation between SDI and ASMR in 21 regions (B) and 204 countries (D).

Supplementary Figure S2. Predicting ASR and cases in high SDI areas under 5 years old and over 70 years old for the next 10 years. Predictive analysis of ASDR, ASMR, the number of DALYs, and the number of death in children under 5 years old in high SDI (A). Predictive analysis of ASMR and the number of death (B), ASDR and the number of DALYs (C) in elderly over 70 years old.

Supplementary Figure S1

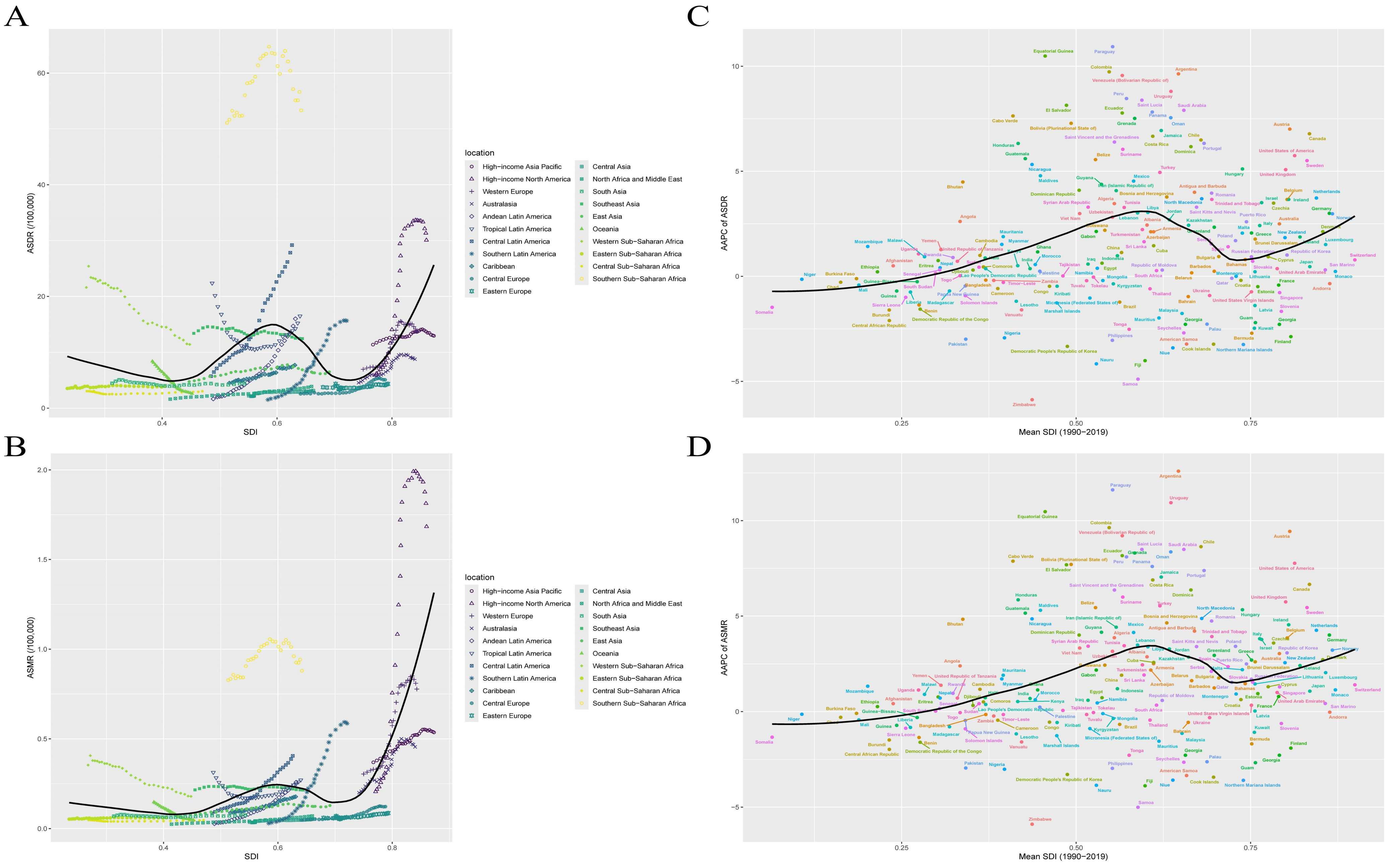

Supplementary Figure S2

A

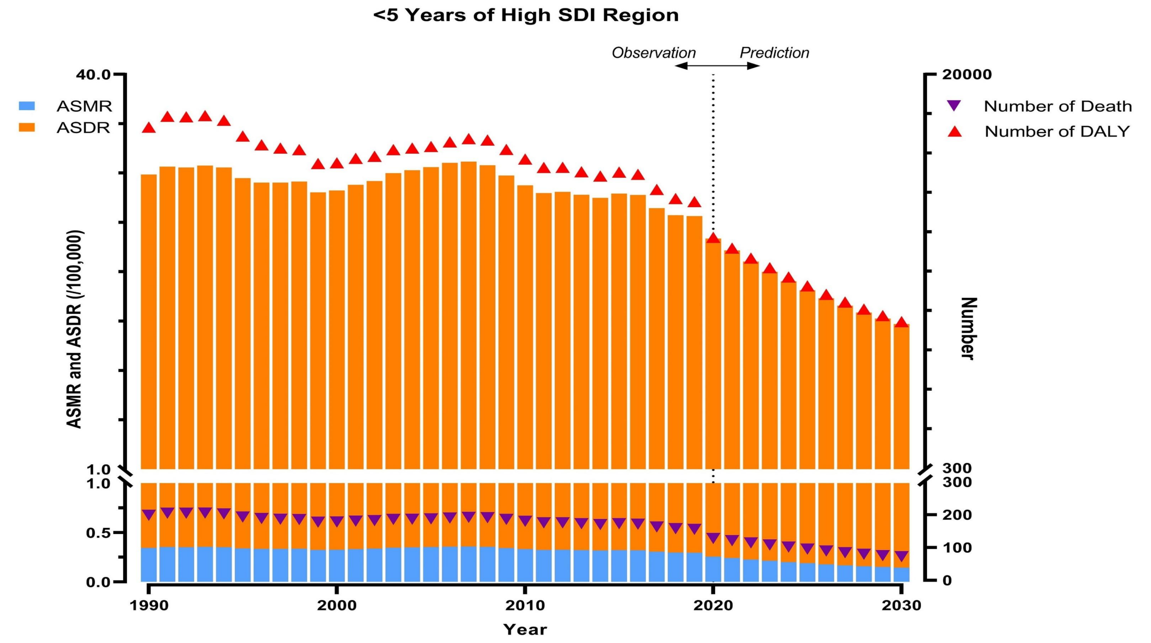

B

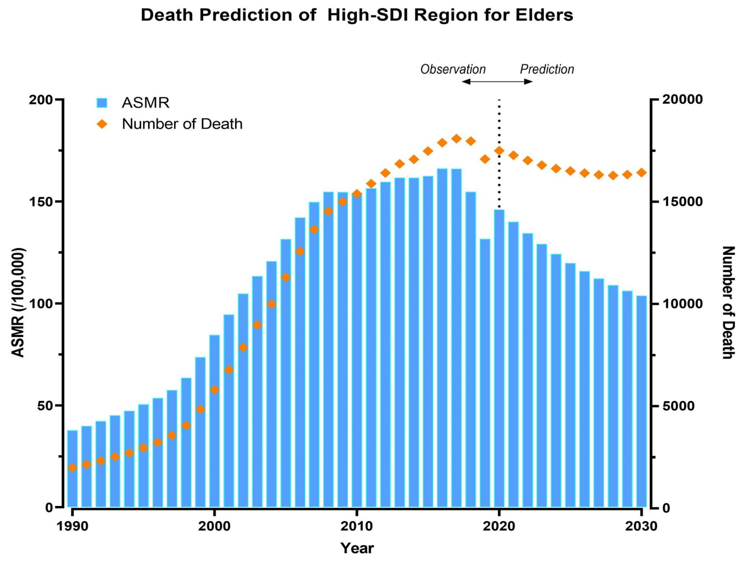

C

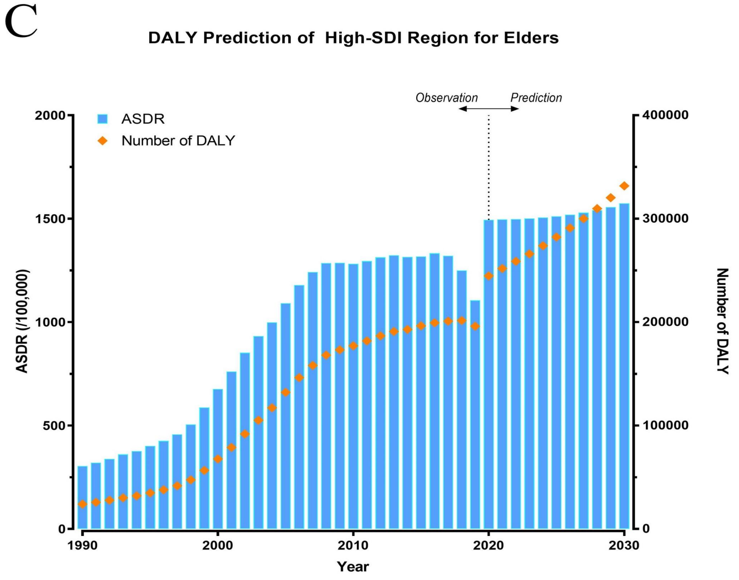

Supplement: Supplemental material — Supplemental information for SDI related analysis and disease prediction. [file spectrum.01290-24-s0002.pdf]
